# Antiretroviral Treatment in HIV-1-Positive Mothers: Neurological Implications in Virus-Free Children

**DOI:** 10.3390/ijms18020423

**Published:** 2017-02-15

**Authors:** Antonio Victor Campos Coelho, Paola Maura Tricarico, Fulvio Celsi, Sergio Crovella

**Affiliations:** 1 Department of Genetics, Federal University of Pernambuco, Avenida da Engenharia, Cidade Universitária, 50740-600 Recife, Brazil; avccbio@gmail.com; 2 Institute for Maternal and Child Health, Scientific Institute for Research, Hospitalization and Care (IRCCS) Burlo Garofolo, 34137 Trieste, Italy; tricaricopa@gmail.com (P.M.T.); fulvio.celsi@gmail.com (F.C.); 3 Department of Developmental and Reproductive Sciences, Genetic Unit, University of Trieste, 34127 Trieste, Italy

**Keywords:** antiretroviral therapy, neurological impairment, neuro-inflammation, autophagy, epigenetic mechanisms

## Abstract

Since the worldwide introduction of antiretroviral therapy (ART) in human immunodeficiency virus type 1, HIV-1-positive mothers, together with HIV-1 testing prior to pregnancy, caesarian birth and breastfeeding cessation with replacement feeding, a reduction of HIV-1 mother-to-child transmission (MTCT) has been observed in the last few years. As such, an increasing number of children are being exposed in utero to ART. Several questions have arisen concerning the neurological effects of ART exposure in utero, considering the potential effect of antiretroviral drugs on the central nervous system, a structure which is in continuous development in the fetus and characterized by great plasticity. This review aims at discussing the possible neurological impairment of children exposed to ART in utero, focusing attention on the drugs commonly used for HIV-1 MTCT prevention, clinical reports of ART neurotoxicity in children born to HIV-1-positive mothers, and neurologic effects of protease inhibitors (PIs), especially ritonavir-“boosted” lopinavir (LPV/r) in cell and animal central nervous system models evaluating the potential neurotoxic effect of ART. Finally, we present the findings of a meta-analysis to assess the effects on the neurodevelopment of children exposed to ART in utero.

## 1. Introduction

The reduction of worldwide human immunodeficiency virus type 1 (HIV-1) mother-to-child transmission (MTCT) in the last few years represents one of the most successful preventive interventions in fighting a substantial health problem. This success has been possible thanks to the introduction of HIV-1 testing prior to pregnancy, caesarian birth and avoiding breastfeeding, in parallel giving alternatives to HIV-1-positive mothers (maternal milk banks, for example) to safely feed newborns.

Worldwide HIV-1 MTCT prevention programs, such as the United Nations Millennium Declaration [1,2,3] strongly contributed to this successful intervention.

Unfortunately, underdeveloped regions of the world still have quite high rates of HIV-1 MTCT (including Nigeria, 23%, Angola, 21%, Chad, 19%, Ivory Coast, 16%, and Democratic Republic of Congo, 15%) [2], thus requiring improvement of the measures for prevention. As an example, in some countries of Sub-Saharan Africa, as well as in the Indian sub-continent and some regions of Southeast Asia, the coverage of pregnant women who receive antiretroviral therapy (ART) HIV-1 MTCT prevention is under 41%, a very low rate when compared to countries such as Brazil, Uruguay Thailand, South Africa, Namibia, and Mozambique, which all have coverage over 95%.

Epidemiological data report that new HIV-1 infections in children have been reduced by 48% between 2009 and 2014 in 21 African priority countries due to ART coverage (World Health Organization, WHO) [3]. Consequently, an increasing number of children born to HIV-1-positive mothers are being exposed in utero to ART. A systematic review of pharmacokinetic data pertaining to placental transfer of antiretroviral drugs observed that the nucleoside analog reverse transcriptase inhibitors (NRTIs) abacavir (ABC), lamivudine (3TC), tenofovir (TDF) and zidovudine (AZT), the non-nucleoside analog reverse transcriptase inhibitors (NNRTIs) efavirenz (EFZ) and nevirapine (NVP), and first-line protease inhibitors (PIs) such as ritonavir-“boosted” lopinavir (LPV/r) and atazanavir (ATV/r) reliably cross the placental barrier, reaching neonate blood concentrations similar to maternal blood levels [4]. This being so, questions are now rising about the effects of this exposure.

Since the WHO is actively developing interventions for prevention of HIV-1 MTCT, envisaging the administration of ART to HIV-1-positive women worldwide (WHO Option B+) [3], we can hypothesize an impact of this ART treatment in terms of adverse effects on HIV-1-free children as the price paid to decrease rates of HIV-1 MTCT.

For this reason, surveillance of adverse effects in children born to HIV-1-positive mothers treated with antiretroviral drugs is strongly needed. The recent study by Williams et al. for example, describes the surveillance for ART toxicity in 22 USA clinical sites, reporting adverse effects such as metabolic disorders related to AZT use. In this study, 12.4% of metabolic disorder cases were reported in cases of exposure to AZT in-utero versus 6.6% of AZT-unexposed cases, yielding an adjusted relative risk of 1.69 and a 95% confidence interval of 1.08 to 2.64 with a sample size of 1524 children—79.6% of which were exposed to AZT in-utero. The authors also investigated neurologic and language disorders, but after adjustment for other factors (mother age, race, pre-pregnancy body mass index and pre-existing health conditions, among other characteristics), there was no association of any ART regimen with neurological impairments in the children [5].

Therefore, we will review the following topics: the drugs commonly used worldwide for HIV-1-MTCT prevention, clinical reports of ART neurotoxicity in children born to HIV-1-positive mothers, and neurologic effects of LPV/r in cell and animal central nervous system (CNS) models for evaluating the potential neurotoxic effect of ART. Additionally, we performed a meta-analysis to assess ART effects over the neurodevelopment of children exposed to ART in utero.

## 2. Antiretroviral Drug Regimens for HIV-1 Mother to Child Transmission Prevention

Most MTCT happens during birth or through breastfeeding, therefore, starting ART pregnancy is paramount to MTCT prevention. An effective combination ART regimen starting around 28 weeks of pregnancy and lasting through to 14 days post-partum assures viral suppression and can reduce HIV-1 MTCT rates to 1% or less from a range of 15% to 45% without any intervention [3,6,7].

Since more women have been covered by ART in the past few years, the safety profile of antiretroviral drugs during pregnancy has been closely followed by clinicians and researchers. As such, the most recent USA National Health and Human Services Department (HHS) guidelines have been updated to promote more straightforward conduct. Women that are already receiving treatment are now allowed to retain the same regimen they have been receiving if viral suppression has been attained and persists throughout the whole pregnancy [8]. On the other hand, if the pregnant woman is ART naive, the treatment start regimen choice closely follows the general guidelines for USA adults living with HIV-1. The preferred starting regimen should consist of a NRTI “backbone” of ABC and 3TC or TDF with 3TC or emtricitabine (FTC), plus a third drug from the PI class (ATV/r being the preferred choice) or an integrase inhibitor. Alternative choices for first-line treatment include AZT and 3TC as an alternative NRTI, LPV/r as a PI alternative, or a regimen including the NRTI backbone plus a NNRTI, substituting the PI altogether. EFZ is the primary option in this case [8].

These recommendations have been based on the pharmacokinetics properties of the drugs, which are altered by pregnancy; in fact, some drug-metabolizing enzymes have an apparent decrease or increase in activity. Thanks to these considerations, the LPV/r dosage was increased during pregnancy [9]. AZT plasma levels decrease during pregnancy [10,11] and TDF plasma levels decrease during the third trimester compared with post-partum period [12]. EFZ was deferred as an alternative drug due to potentially severe neurological adverse effects and to account for cases in which in PI use would be contraindicated due to interactions with comedication [8]. Previous teratogenicity concerns arose from primate studies, and there are no available direct data from human studies. However, meta-analyses of the literature have shown that first-trimester EFZ exposure is not associated with higher risk to teratogenicity [13]. Possibly following the USA’s HHS panel updates, Brazil’s Ministry of Health recently issued a similar update to its guidelines. However, the preferred first-line regimen for Brazilian ART naïve adults, including pregnant women, was TDF + 3TC and EFZ, because it is available in once-daily fixed dose combination, which helps treatment adherence. It is also effective against hepatitis B virus (HBV) co-infection (if any) and has a lower potential for hematological toxicity and lipodystrophy than AZT. TDF + 3TC and LPV/r is the immediate alternative regimen.

Given the relative “novelty” of these guidelines, it is probable that there still are women receiving LPV/r-containing regimens in Brazil and other countries, and as the guidelines are being progressively adopted, EFZ use during pregnancy may increase soon. As mentioned before, neither LPV/r nor EFZ cause teratogenicity during pregnancy. However, eventual long-term effects of ART in terms of neurodevelopment are not fully elucidated and need further investigation.

## 3. Antiretroviral Therapy Adverse Effects in the Context of HIV-1 Mother to Child Transmission Prevention

As mentioned before, pregnancy promotes physiological changes that must be taken in consideration during ART administration since they influence antiretroviral drug pharmacokinetics [14], which can be related to occurrence of adverse effects and loss of viral suppression.

The short-term adverse effects, relating to events that happen shortly after initiation of ART, include low birth weight, hematologic toxicity, liver dysfunction, myopathy, cutaneous rash or even toxicity-related deaths; longer-term adverse effects that include mitochondrial dysfunction and neurotoxicity are a matter of debate.

To date, little information regarding the longer-term adverse effects is available. Poirier and collaborators observed a long-term effect of AZT in children born to HIV-1-positive mothers. They detected mitochondrial DNA depletion, a marker of mitochondrial dysfunction, in the leukocytes of children. This was present at birth and persisted up to two years of age [15]. Subsequently, other studies confirmed mitochondrial dysfunction as an important long-term effect of AZT alone and in combination with other nucleoside analogues that can manifest as a variety of clinical and laboratory abnormalities, including hematologic and liver function, myopathy, and CNS disorders [16,17]. Of utmost concern is the possibility that antiretroviral drugs could cause damage to the fetus, since drugs and other substances are typically transferred through the placenta to the fetus [18]. Blanche and colleagues [19] for example, described the effect of AZT on mitochondria and related them to neurological malformations, characterized by cortical and subcortical brain atrophy and hypersignaling of white matter, as well as neurologic-related symptomatology in newborns. Rice and collaborators observed that in utero ART exposure was associated to a higher risk of language delay observed in one-year-old HIV-1-exposed uninfected infants [20]. However, there is no clear quantitative evaluation of these defects nor a correlation with the type of ART used. In addition, some authors reported an association between ART and delayed infant growth (mean weight-for-age z-scores: −0.53 in ART-exposed in utero versus −0.30 in unexposed children, total sample size of 819 children, 516 of them ART-exposed) [21].

Recent studies addressed two issues that are debated. The first issue is teratogenicity of antiretroviral drugs. A meta-analysis that included 23 studies conducted across the world, totaling 2026 live births between 1986 and 2013 from women that received EFZ during the first trimester of pregnancy, found a pooled prevalence of 1.63% of congenital defects (44 live births), with only one being a neural tube defect (prevalence of 0.05%, close to the expected in the general population). Therefore, EFZ use was not associated with an increased risk to birth defects, but more live birth surveillance is warranted.

A study conducted in France which included 13,124 births between 1994 and 2010, found an association between AZT and a slightly higher risk of heart defects (prevalence of 2.3% in the children of women that received AZT during the first trimester versus 1.1% from women who did not, with an adjusted odds ratio of 2.2). The authors did not find a significant association between EFZ, LPV/r, NVP, TDF or ABC and higher risk to congenital malformations during primary analysis; during a secondary analysis with a different classification scheme, they found an association between EFZ use during the first trimester with the occurrence of four children born with neurological malformations. However, they were not neural tube-related, two of the defects may have no clinical significance and each had distinct embryologic origins [22].

The second issue is preterm delivery. There is a debate whether ART are associated with premature birth (usually defined as a birth that occurs before 37 gestation weeks), with recent reports indicating there is indeed an increased risk for preterm birth. A recent systematic review with a meta-analysis from 10 published studies carried out between 2002 and 2013, totaling 23,490 births, concluded that PI exposure during pregnancy is associated with a slightly higher risk of preterm delivery (pooled odds ratio = 1.32 with 95% confidence interval = 1.04–1.59). Preterm birth is strongly associated with future complications in the child’s life, such as the possibility of cerebral palsy, difficulties with motor skills, lower cognitive performance and learning disabilities.

It is unknown if these drugs exert any influence that is not immediately apparent, in contrast with congenital malformations and preterm delivery. They may be just two of the possible effects in the antiretroviral drug-related adverse effects spectrum. The development of the central nervous system of the children protected against MTCT may be influenced in more subtle ways, since the developing CNS is a structure with great plasticity and after the peripheral nervous system, it is the most affected by mitochondrial disorders, which may cause induce epilepsy, stroke-like episodes, ataxia, spasticity, extrapyramidal abnormalities, bulbar dysfunction, psychiatric abnormalities, neuropsychological deficits, and hypophysial abnormalities. For this reason, surveillance of adverse effects in children born to HIV-1-positive mothers receiving ART is strongly needed, and it is being applied in both developed countries (USA) [5] and resource-limited settings (Botswana, South Africa, Malawi, Mozambique, Burkina Faso and Kenya) [23].

Therefore, some authors have been addressing aspects of the neurodevelopment of HIV-1 exposed but uninfected children.

## 4. Neurodevelopment in HIV-1- and ART-Exposed but Uninfected Children: A Meta-Analysis

The Pediatric AIDS Clinical Trials Group Protocol 076 (PACTG 076) was the hallmark that HIV-1 MTCT prevention was possible, demonstrating that the transmission rate was almost 70% lower if the woman had received AZT rather than placebo. Soon after, concerns regarding AZT safety, both during pregnancy and over the life of the child, emerged. A subsequent study aimed to “evaluate the long-term effects of in utero exposure” to AZT in children from the PACTG 076. The authors examined 234 uninfected children born to HIV-1-infected women that received AZT during pregnancy, as designed by the protocol. They assessed, among other clinical parameters, the cognitive and developmental function through Bayley Scales of Infant Development second edition (BSID-II) and concluded that there were no significant differences between children exposed to AZT in utero and AZT unexposed children regarding neurologic development and function.

We performed a literature search to address this issue with PubMed and MeSH databases using keywords such as “antiretroviral therapy, highly active”, “pregnancy”, “children” and “adverse effects” along with generic names of antiretroviral drugs (“zidovudine”, “efavirenz”, “lopinavir” and so on) published after 1996 (the year of the inception of highly active ART as we know it). We found 380 unique studies and selected for review only studies involving HIV-1-exposed uninfected children that were exposed to antiretroviral drugs in utero, which underwent neurological evaluations. We found that other studies already had performed similar reviews of this topic [24,25], reaching the same conclusions that ART does not have a strong effect on neurodevelopment in children. Therefore, we decided to perform a meta-analysis focusing the comparison of neurodevelopmental functions assessments of HIV-1 and ART exposed in utero uninfected children and control children (ART unexposed), thus ruling out any effect of the virus over the CNS (and consequently isolating the eventual effects of the in utero ART exposure). We selected the studies cited by Ngoma et al. [25] and added data from other studies [26,27]. This resulted in six studies that assessed neurodevelopmental functions through BSID-II [26,27,28,29], BSID third edition (BSID-III) or Full-Scale Developmental Quotient (FSDQ) [25,30]. Another five studies reviewed were not included in the meta-analysis because, although they involved neurodevelopmental evaluation in children in the context of HIV-1 MTCT, we could not ascertain if recruited HIV-1-infected mothers received ART in four studies and could not extract data from the remaining one, as there was no clear quantitative evaluation of these defects or the association with the type of ART used [20].

The studies recruited a total of 2210 HIV-1- and ART-exposed in utero uninfected children and 414 control children (ART unexposed in utero), with ages ranging between three and 36 months. We extracted psychomotor development index (PDI) or similar scoring component data from each study. If the study assessed the neurodevelopment status at several timepoints, we extracted the scoring from the last one. Psycho Scorings were pooled through a random-effects model with standardized mean differences (SMD) for continuous outcomes, especially suited to outcomes measured with different scaling schemes [31], the FSDQ, and the BSID-II and III as mentioned above. Table 1 summarizes the characteristics of the studies.

We chose a random effects meta-analysis model because we considered that since the children were exposed to different antiretroviral drugs across studies, the magnitude of their effect over neurodevelopment could, in theory, also vary across studies. Heterogeneity among sample sizes was quantified by *I*^2^ measure. Statistical significance was set at 0.05 level with the null hypothesis that the pooled SMD was zero (in other words, true neurodevelopmental scores did not differ between ART-exposed and ART-unexposed children). The meta-analysis and associated 95% confidence intervals (CI) were performed through “meta” package [33] for R software version 3.3.1 [34].

The heterogeneity among the six studies was quite high (I2 = 72.8%, 95% CI = 37.4%–88.2%), reflecting the sample size variability. The pooled SMD was very low (−0.04; 95% CI = −0.3–0.2, *p* = 0.78), meaning that ART-exposed children scored negligibly below ART-unexposed children. Thus, ART alone does not seem to be associated with important consequences to neurodevelopment.

Unfortunately, most studies included in the meta-analysis did not report the exact timing of ART exposure, except for one study. Williams et al. [29] reported that they ascertained the trimester of in utero exposure for 1614 children and observed that mean mental development index (MDI scores, component of the Bayley scales) were marginally higher for second-trimester exposure and significantly higher for third-trimester exposure, as compared with those who were not exposed during that respective trimester. Therefore, more studies are needed to assess the impact the timing of ART exposure over neurodevelopment of ART-exposed children.

Additionally, it is important to consider maternal substance abuse along with antiretroviral drugs. As observed in one of the included studies, a prospective cross-sectional Canadian study involving 39 HIV-1-exposed but uninfected children (18 to 36 months of age) whose mothers had received ART for at least 1 week during pregnancy and AZT during delivery, maternal substance abuse had a stronger effect on BSID-II indexes (they were lower when compared to controls) than ART per se, possibly because it is strongly associated with preterm birth. However, the frequency of prematurity was not statistically different when comparing the two study groups. They did not elaborate on the effect of PI choice, in which nelfinavir was apparently the only one used in their sample (used by 13 mothers, 33.3%).

Therefore, maternal substance abuse together with antiretroviral drugs may synergize their adverse effects, causing more risk to preterm birth, which is linked to neurodevelopmental delays, as discussed in the previous topic. It is important to mention that maternal substance abuse is just one factor among several other socioeconomic factors which play an important role in birth outcomes. In other words, socioeconomically disadvantaged women not only tend to use more licit and illicit drugs, but also tend to have lower nutritional status and have less access to prenatal care (more common in African countries), which are risk factors for preterm birth [35].

More studies are needed to understand the interplay between ART exposure and its timing, maternal substance abuse, preterm birth and their consequences on the neurodevelopment of children. Meta-analyses as the one presented here are valuable tools to discover confounding factors and confirm the safety (or risk) of present and future antiretroviral drugs, contributing to further improvement of HIV-1 MTCT prevention strategies and ensuring the best quality of life to future mothers living with HIV-1 and their children.

## 5. Cellular and Animal Models of Antiretroviral Therapy-Related Central Nervous System Adverse Effects

In adult patients, HIV-1-associated neurocognitive disorder (HAND) is a condition present in almost 50% of individuals undergoing ART. HAND syndromes are characterized by cognitive, motor and behavioral changes which can heavily impact the quality of life of patients [36]. Thanks to introduction of ART neurological symptoms associated with HIV-1 infection are largely diminished. Since ART is a lifelong therapy, in recent years different studies have analyzed pharmacokinetics of ARTs in the CNS, giving rise to a CNS-specific penetration effectiveness (CPE) index, which is the ratio of the plasma and cerebrospinal fluid concentrations of a specific drug. The CPE index is an important indicator, but is not suited to suggest the occurrence of neurotoxic effects. Indeed, different cohort studies have analyzed if a possible correlation exists between ARTs with low CPE and HAND syndromes, but with conflicting results and without considering the milder forms of HAND, like mild cognitive impairment (MCI). In the case of ART regimes with high CPE an increase in HAND prevalence has been observed, suggesting a possible link. However, up until now, a strong correlation has not been demonstrated [37]. It is also important to notice that CPE score has not been evaluated for pediatric patients and thus it is not possible to determine how antiretroviral drugs penetrate a developing brain. In this context, cellular models, firstly, and animal models, secondly, remain fundamental instruments to assess the neurotoxicity of these drugs.

### 5.1. Neuronal Cell Models

Primary neuronal cell cultures from either mice or rats are widely used to model different CNS aspects, from development to mechanisms of CNS diseases. These cultures provide a simplified model of the CNS, as they can provide important insight for intracellular and intercellular environments. Using primary rat forebrains cells, Robertson and co-workers [38] analyzed the toxic effects of 15 different antiretroviral drugs; they evaluated parameters such as neuronal morphology and sensitiveness to glutamate. The results show that at least some of them (ABC, EFZ, NVP and ATV) possess high toxic effects, although at concentrations seldom used in clinical practice. Another study employed primary rat cortical neurons to assess their toxicity, using ritonavir, saquinavir and AZT in therapeutic concentrations. The authors found that these three antiretroviral drugs are toxic in their experimental conditions.

The clear majority of studies involving neurotoxicity of antiretroviral drugs are however focused on neuropathies and thus primary sensory neuron or dorsal root ganglion (DRG) neuronal cultures are very often used. In human DRG cultures, different NRTIs (such as didanosine, zalcitabine, stavudine and AZT) have been shown to reduce axonal length and similar findings were obtained using rat DRG cultures. This kind of cell model revealed its usefulness in describing mechanism of neuropathy induced by these drugs, but it still limited in interpretation of CNS neurotoxicity.

Cell lines were also used to assess neurotoxicity and several studies have used the SH-SY5Y, a well-known neuroblastoma cell line, prevalently used for modeling Parkinson’s or Alzheimer’s disease. Some recent studies describe toxicity of EFZ with clearly described neurological side effects [39]. These works delineate the specific mitochondrial toxicity of EFZ, with reduction of mitochondrial membrane potential and activation of macroautophagy [40,41,42] providing a possible link with neurological impairment observed in patients under ART. Neurotoxic effect of LPV/r was recently tested also in SH-SY5Y cells, demonstrating that combination of these two drugs can induce apoptotic cell death in concentration comparable to therapeutic administration. It has been reported that LPV/r induced mitochondrial damage, increase of heme oxygenase RNA expression levels and reactive oxygen species (ROS) generation, followed by apoptosis, corroborating previous evidence that oxidative stress is a key event in causing PI-related adverse effects. Apoptosis following mitochondrial damage seem to play a pivotal role in side-effects of ART [43]. The mechanisms by which the HIV-1 drugs induce mitochondrial dysfunction are not clearly established. A mechanism may be related to insulin resistance; in fact, reduced glucose uptake following exposure to PIs might trigger mitochondria stress. Supporting evidence is that individuals living with HIV-1 with metabolic dysfunction have a significantly higher risk of developing neurologic complications [44].

### 5.2. Astrocytes Cell Models

The role of astrocytes is to support and sustain neurons, modulating neurotransmitter release and recycling.

A limited number of studies have used primary astrocyte cell cultures to assess ART toxicity in CNS. Human fetal astrocytes (HFA) and progenitor-derived astrocytes (PDA) have been used to demonstrate that under inflammatory conditions they can become permissive to HIV-1 infection, transforming the current view about the role of astrocyte in virus reproduction in CNS [45]. Recently, toxicity of protease inhibitors (PIs) amprenavir and LPV were investigated in HFA, demonstrating that treatment with therapeutic concentrations of those PIs disrupts glutamate metabolism in those cells, thus lowering threshold for excitotoxicity.

Glial-derived cell lines (U-251MG) have been used in previous studies [41,42] to assess EFZ toxicity. The first work demonstrates that EFZ induces expression of an inducible isoform of nitric oxide (NO) synthase, followed by NO-mediated mitochondrial damage, interfering with complex IV of the mitochondrial electron transfer chain (mETC). Moreover, EFZ can block activity of complex I of mETC, demonstrating a “double hit” action in respect of mitochondrial membrane potential. The second also analyzes mETC, expanding the previous findings and showing that EFZ increases proton leakage from the mETC and reduces oxygen consumption. In summary, EFZ damages mitochondrial activity, lowering metabolism and rendering cells more prone to apoptotic cell death in concentrations similar to the ones found in cerebrospinal fluid.

### 5.3. Oligodendrocyte Cell Lines

Oligodendrocyte cell culture is used as a model of myelin sheath generation and how it is involved in various pathologies [46,47,48]. Considering that abnormalities in white matter is one of the typical markers of HAND, it is of interest to analyze effects of antiretroviral drugs in these cells. Currently, only one study approached the effect of LPV/r on oligodendrocytes, showing that these drugs, at therapeutic concentrations, impair maturation of murine myelinating cells [49]. These results suggest that PI administration could have a profound impact in infant and child CNS development, since cortical myelination is a continuous process until late adolescence.

### 5.4. Animal Models

The focus of our review is to discuss ART side effects in uninfected children born to HIV-positive mothers treated with ART. This being so, we report some animal models used to evaluated the effects of HIV infection and ART on CNS of uninfected animals.

The HIV-1 research field has benefited from the use of different animals models; in particular, as simian immunodeficiency virus (SIV) is the ancestor of HIV-1 [50], primates have been widely used as the model of choice to a variety of HIV-1-related research.

Indeed, SIV has a similar pathogenesis to HIV-1, with infection of CD4^+^ cells and macrophages, immune suppression and neurological damage; moreover, SIV is also transmitted from the mother to child in similar ways to HIV-1, increasing the importance of non-human primate models.

As reviewed by Carryl and co-authors, [51] there are different primates models recapitulating pediatric infections. *Macaca nemestrina*, the pigtail macaque, can be infected with HIV-1 (specifically HIV-2_287_) and used to model MTCT. Infected infant macaques performed poorly in cognitive and motor tests, with poorer performance correlated with decreased CD4^+^ counts. These neurobehavioral deficits are closely similar to those founds in pediatric HIV-1 patients [52]. Another macaque species, *Macaca mulatta*, the rhesus macaque, has been widely used for modelling pediatric HIV-1 infection; these old world monkeys can be infected with three SIV strains (SIVmac239, SIVmac239/316, and SIVmac251) that can penetrate the CNS. Infant macaques infected with these virus strains develop CNS lesions, with lymphocyte infiltration in cortical gray and white matter and basal ganglia [53]. Recently, a systematic histopathological examination of SIVmac251-infected macaques’ brains demonstrated a specific loss of pyramidal neurons in hippocampal areas; moreover, immature neurons in dentate gyrus were also lost [54]. This model closely recapitulates some features found in pediatric HIV-1 infections, suggesting its use for studying HIV-1 infection effects and ART aspects.

The SIVmac251 strain has been used for evaluating the effects of two drugs: AZT and TDF. Two studies demonstrated that AZT treatment pre or post SIV exposure reduces CNS damage [55,56] and treatment with TDF improved survival and ameliorated CNS damage with high efficacy [57].

ART neurological effects have been described in *Erythrocebus patas*: pregnant uninfected animals were exposed to AZT and 3TC at equivalent doses to those administered in humans, causing lowering of mitochondrial DNA content and change in mitochondria morphology in brain and liver of some specimens at birth and after one year of age [58]. Interestingly, ART treatment in *Macaca nemestrina* induces lowering of synaptophysin and calcium/calmodulin-dependent protein kinase II (CamKII) levels, two neuronal cells markers, demonstrating a possible direct effect of those drugs on neuronal cells [59]. These two studies demonstrate usefulness of non-human primates as models for ART-induced neurotoxicity and further studies are thus needed to better assess the potential toxic damage to the CNS caused by antiretroviral drugs.

However, working with these animals is costly and time-consuming; and for these reasons murine and rats model have been developed. One of the most crucial limitation in use of these models in HIV-1 research is the fact that both mice and rats are not natural hosts of HIV-1 nor do they naturally get infected by HIV-1 [60]. Strategies have been developed to study HIV-1 pathogenesis in rodents, although they require either genetic manipulation of mice or intracranial administration of Tat/gp120 viral proteins [61]. Of these models, only the latter has been used as a model for pediatric HIV-1 infection. Neonatal intrahippocampal injection of Tat_1-72_ alone or in combination with gp120 causes selective neuronal loss in specific hippocampal regions: gp120 causes neuronal loss in CA2/3 regions, while Tat_1-72_ provokes neuronal damage and increases number of astrocytes and of oligodendrocytes in dentate gyrus region [61]. Injecting Tat in mice as early as in the first post-natal day causes spatial memory and sensorimotor gating impairments, typical signs of different cognitive problems [62]. In conclusion, these models recapitulate some features found in HIV-1-infected children and indicate the crucial role of Tat protein in pediatric neuronal damage. However, it possesses an intrinsic weakness: since it is not an infection model, it therefore does not originate typical features of neuroinflammation, such as interleukin 1 β (IL-1β) secretion.

In 2001, an HIV-1 transgenic rat (HIV1-Tg) was established as a novel model for studying HIV-1 pathology. This animal model has been engineered to express the entire viral genome, except gag and pol genes, blocking viral replication. Viral proteins are expressed in almost all tissues, including brain, lymph nodes, thymus, liver, lung, kidney, spleen, epidermis and blood [63]. These rats are phenotypically indistinguishable from wild type animals until five or six months of age, when they start to develop AIDS-related pathologies until death around nine or 10 months.

This model could be a useful tool to investigate toxicity of ART in a context of HIV-1 infection and possible drugs to enhance cognitive functions in individuals living with HIV-1.

Models like this helped to describe neurologic side effects for LPV/r. Adult male C57BL/6 mice treated with clinically relevant doses of LPV/r during four weeks presented cognitive impairment, cerebrovascular injury associated with decreased synaptic markers, increased inflammation levels, and cerebral cortex vulnerability. Metabolic disturbances such as lipodystrophy, insulin resistance and hyperlipidemia were also observed, therefore, supporting the evidence that metabolic dysfunction could mediate adverse neurologic effects [64,65]. In fact, new compounds able to prevent metabolic dysfunction are being studied [65], and they may bring benefits for individuals receiving PIs.

In conclusion, we presented a variety of cellular and animal models employed to study the interplay between HIV-1 infection, CNS and antiretroviral drugs; these models are useful tools to examine effects on CNS generated by continuous administration of ART and to determine ways to improve neurological symptoms experienced by patients.

## 6. The Role of Epigenetics in Antiretroviral Drug-Related Adverse Effects

A new concept that has been emerging in recent years is the link between epigenetic consequences and adverse effects of antiretroviral drugs. Epigenetics involves the regulation of gene expression without any changes to the DNA sequence itself. Epigenetic mechanisms play an important role during the HIV-1 infection, mediating the integration of the HIV-1 into the host’s genome, a crucial step in the viral life cycle [66]. There is evidence that aggravated neurodegenerative, cognitive, motor and behavioral symptoms observed in persons living with HIV-1 are connected to epigenetic and transcriptional changes, and substance abuse may exacerbate these changes (such as methamphetamine) [67]. To date, only the epigenetic effects of AZT were studied; Nyce and collaborators showed that AZT induced chromatin hypermethylation [68]. Moreover, there is a report that organization of constitutive heterochromatin in leukocytes of both myeloid and lymphoid lineages of children born to mothers infected with HIV-1 were altered by the AZT that they received during the pregnancy [69]. Unfortunately, there are no data regarding the epigenetic effects of other ART, but the impact of ART used in HIV-1-MTCT in terms of epigenetic changes in children born to HIV-1-positive ART-treated mothers should be investigated in the very near future: the possibility of epigenetic changes transmitted to subsequent generations is an issue that should be considered.

## 7. Future Perspectives in the Management of Treated Children Born to HIV-1-Positive Mothers

The surveillance of ART short, medium and long-term effects on children born to HIV-1-positive mothers is becoming essential, due to the previous reports about adverse effects in children [70,71]. This surveillance can be performed with different strategies, depending on health center/hospital resources that take care of HIV-1-positive mothers and their children.

Independently of the resources available, a questionnaire should be administered to know the clinical, socioeconomic and environmental characteristics of the mothers. Based on two studies performed in USA [5] and South Africa [72] (high and low/medium income countries, respectively), we suggest a minimal set of data to be collected. Merging the indications from the two studies, the following items should be considered: demographic characteristics, ethnicity, schooling level, family income, history of diseases present in the mother’s family (including viral co-infections, history of eventual hospitalizations), mothers’ ART regimens and time of exposure, history of substance abuse, tobacco and/or alcohol consumption, total time in prenatal care, type of delivery (caesarian section for HIV-1 MTCT prevention is to be expected), occurrence of preterm birth, and postpartum examination of children (appearance, pulse, grimace, activity, respiration (APGAR) score, weight, length, head circumference etc.). Following the questionnaire data collection, neurocognitive, locomotors and neurological exams should be performed.

At present, many HIV-1 mother care centers are promoting clinical and follow-up activities of both mothers and children at the same time, thus reducing data dispersion and optimizing time and resource availability. It is valuable to consider that low income countries, where fewer HIV-1 care centers are available and are sometimes located far away from the patients’ residence site, it is more convenient to schedule all follow-up appointments in a single day to reduce the costs for the families attended; this greatly improves adherence and reduces the number of losses to follow-up. Social programs may cover transportation costs, provide food stamps or supplemental nutrition programs, for example, to retain low-income families in healthcare.

The first neurobehavioral observation can be performed during the neonatal period using the very simple and efficient General Movements (GMs) test [72,73], which can be used in HIV-1 reference centers independently of their income; this test allows rapid identification of neurobehavioral issues and is considered suitable for successful rapid neuropsychiatric intervention [74].

The GMs method consists in filming the spontaneous motor skills of infants to observe eventual atypical motor patterns that literature relates with motor and neurological pathologies. The GMs method is known worldwide to be inexpensive since it just requires standard video cameras, web-cams, and videocameras to register the movements of newborns, thus costing less than $1 USD per patient. GM observations can be applied either in neonatology wards (preterm newborns, for example) or on beds, with the newborn awake and in prone position. GM evaluation requires trained personnel (neurologist/therapist) for observation; the use of telemedicine tools could successfully support parents’ care [75].

In Brazil, our research group has a research partnership with Instituto de Medicina Integral Professor Fernando Figueira (IMIP), a reference healthcare center for mothers and their children in Recife (Pernambuco state, Northeast Brazil) which developed telemedicine applications.

We intend to take advantage of IMIP’s infrastructure and trained personnel to follow up on children born to mothers living with HIV-1. Their neurodevelopment will be measured using the Bayley test, third edition. According to the obtained results, corrective measures can be adopted and their impact evaluated in future appointments.

The proposed follow-up strategy would permit a comprehensive long-term evaluation of the in utero ART effects on ART-exposed children. It will also permit the recommendation of rehabilitation and the application of corrective measures to alleviate eventual neurobehavioral defects, tailoring support to children in an age-specific manner according to their needs, such as schooling etc.

We are aware that we are proposing just one hypothetical workflow coming from our experience at IMIP, in Brazil. Many other possible follow-up schemes have been proposed so far [76], but no matter which one is adopted, the care of ART-exposed children should be now strongly considered during clinical care strategy development, both in low-medium and high income countries due to the possible effects of ART in utero. Finally, we highlight the need for a global standard for these follow-up activities worldwide.

## 8. Concluding Remarks

The development of ART is undoubtedly one of the greatest achievements in the fight against HIV-1. With it, millions of lives have been saved from AIDS-related deaths and generations of children have been protected against HIV-1 MTCT. The present challenge is to reassure that present (and future) antiretroviral drugs are safe for use during pregnancy. Thus, we reviewed the literature and performed a meta-analysis of studies reporting possible neurodevelopmental side effects in children born to HIV-1-positive mothers treated with ART in order to address this question. The meta-analysis findings did not point to a significant cognitive impairment of children exposed to ART in utero. However, other reports suggest a possible link between ART use during pregnancy with preterm birth, which in turn is associated with a spectrum of neurological issues during childhood. Moreover, ART hematological and mitochondrial toxicity are common, and their occurrence is also matter of attention. Maternal substance abuse, which can potentiate eventual ART adverse effects, should be addressed during follow-up in future studies, so true ART neurological adverse effects can be identified in absence of confounding factors. Cellular and animal neurologic models will remain useful to assess antiretroviral drugs effects at molecular level, helping to shape guidelines for future antiretroviral regimens. Finally, we proposed a follow-up pipeline that we designed to be used in the routine clinical assessment of HIV-1-positive mothers and their children in a third-level pediatric hospital in Northeast Brazil.

## Figures and Tables

**Table 1:** Summary of the six studies characteristics that were included in the meta-analysis for the estimation of the effects of antiretroviral therapy (ART) exposure in utero on neurodevelopmental scores of children born to mothers living with human immunodeficiency virus type 1 (HIV-1).

Reference	Country, Publication Year	Study Type	Scale	Antiretroviral Drugs Received by the Recruited Pregnant Women in the Study	Neurodevelopment Assessment Age (Months)	In Utero ART-Exposed Children			In Utero ART-Unexposed Children			*p*-Value
						Sample Size	Score Mean	Score Standard Deviation	Sample Size	Score Mean	Score Standard Deviation	
[26]	USA, 1999	Prospective cohort	BSID-II	AZT	30	64	101.2	16.6	73	101	21.1	0.84
[27]	Canada, 2006	Cross-sectional study	BSID-II	AZT, 3TC, NVP, NFV	18 to 36	39	93.4	14.1	24	96.6	13.5	Not reported ^a^
[28]	Colombia, 2009	Prospective cohort	BSID-II	AZT, 3TC, NVP, LPV/r, NFV	3, 6, 9, 12, 18 and 24	7	96	15.9	6	103	9.2	0.90
[29]	USA, 2010	Prospective cohort	BSID-II	AZT	6, 12, 18, 24, 30 and 36	1694	92.9	16.9	146	97.9	20.3	0.82
[32]	USA and Puerto Rico, 2013	Prospective cohort	BSID-III	AZT, 3TC, TDF, ATV, LPV/r, NFV	9 to 15	309	102.2	22.9	62	101.8	16.5	0.82
[25]	Zambia, 2014	Cross-sectional study	FSDQ	AZT, LPV/r, NVP	15 to 36	97	101.2	13	103	96.5	11	Not reported ^b^

**A**ZT—zidovudine; 3TC—lamivudine; NVP—nevirapine; NFV—nelfinavir; LPV/r—lopinavir/ritonavir; TDF—tenofovir; ATV—atazanavir; BSID-II—Bayley Scales of Infant Development second edition; BSID-III—Bayley Scales of Infant Development third edition; FSDQ— Full-Scale Developmental Quotient. ^a^ authors reported it was not significant at 0.05 level); ^b^ authors reported a logistic regression of FSDQ < 85 as outcome and controlling by mother income, maternal education level, infant age, and birth weight, resulting in a *p*-value = 0.90).
